# Review of the genus *Hartemita* Cameron, 1910 (Hymenoptera, Braconidae, Cardiochilinae), with the description of six new species from Vietnam

**DOI:** 10.3897/zookeys.102.879

**Published:** 2011-06-02

**Authors:** Khuat Dang Long, Cornelis van Achterberg

**Affiliations:** 1 Institute of Ecology & Biological Resources, Vietnam Academy of Science & Technology, 18 Hoang Quoc Viet Road, Cau Giay, Ha Noi, Vietnam; 2 Department of Terrestrial Zoology, Netherlands Centre for Biodiversity Naturalis, Postbus 9517, 2300 RA Leiden, The Netherlands

**Keywords:** Braconidae, *Hartemita*, new species, key, Oriental, Vietnam

## Abstract

The Oriental and East Palaearctic genus *Hartemita* Cameron, 1910 (Braconidae: Cardiochilinae) is recorded for the first time from Vietnam. Sixteen species of the genus *Hartemita* are currently recognized from Oriental and East Palaearctic regions. One species is newly recorded for Vietnam, *Hartemita singaporensis* (Mao, 1945)and six new species from Vietnam are described and illustrated: *Hartemita coffeana* **sp. n.**, *Hartemita daklaka* **sp. n.**, *Hartemita khuatbaolinhae* **sp. n.**, *Hartemita similis* **sp. n.**, *Hartemita maculata* **sp. n.** and *Hartemita vietnamica* **sp. n.** A key to species of the genus *Hartemita* Cameron is included.

## Introduction

The small genus *Hartemita* Cameron, 1910 (Braconidae: Cardiochilinae) is comparatively rare in collections and easily recognizable by its enlarged hind basitarsus (Figs 5, 22, 55, 83, 100). In this respect they resemble stingless bees (Meliponini) which occur over most of its range and as suggested by one of the referees this may be not coincidence. It has an Oriental and East Palaearctic distribution, but was unknown from Vietnam (Long and Belokobylskij 2003). It comprised 16 species; of these, three are from southern and eastern Palaearctic regions, three are from an intermediate area (Nepal) and the other ten occur in the Oriental region (Dangerfield and Austin 1990, Chou 1995, Chen, He and Ma 1998 and 2004; Belokobylskij and Ku 2001; Ahmad and Shujauddin 2004; Belokobylskij 2005; Yu, van Achterberg and Horstmann 2005). As far as known all species of Cardiochilinae are koinobiont endoparasitoids of lepidopterous larvae. Extensive Malaise trapping in Vietnam resulted in the collecting of one described species of the genus *Hartemita*. Six additional species are new to science and are described in this paper. A comprehensive key to species of the genus *Hartemita* is provided.

## Material and methods

Two recent and larger collections of Cardiochilinae from Vietnam are used for this revision: the Braconidae collection in the Institute of Ecology & Biological Resources (IEBR) at Hanoi (assembled by the first author) and the Netherlands Centre for Biodiversity Naturalis collection (RMNH) at Leiden (assembled during five RMNH-IEBR expeditions in Vietnam).

For recognition of the subfamily Cardiochilinae, see van Achterberg (1993), for a key to the genera of Cardiochilinae, see Dangerfield et al. (1999) and for a diagnosis of the genus *Hartemita*,see Dangerfield and Austin (1990). For the terminology used in this paper, see Dangerfield and Austin (1990) and van Achterberg (1993). The scale bars in the plates indicate 1.0 mm.

## Systematics

### Genus Hartemita

Cameron, 1910

http://species-id.net/wiki/Hartemita

Figs 1 [Fig F2] [Fig F3] [Fig F4] [Fig F5] [Fig F6] [Fig F7] [Fig F8] [Fig F9] [Fig F10] [Fig F11] –86

Hartemita Cameron 1910: 99. Type-species: *Hartemita latipes* Cameron, 1910, by monotypy [examined].
Laminitarsus Fullaway 1919: 57. Type-species: *Laminitarsus muirii* Fullaway, 1919, by monotypy [examined].

#### Biology.

Largely unknown; only one species (*Hartemita buteae*) has been reared from an unidentified Noctuid larva.

##### Checklist and distribution

*Hartemita basilaris* Dangerfield & Austin, 1990, from Indonesia

*Hartemita bruneiensis* Dangerfield & Austin, 1990, from Brunei and East Malaysia

*Hartemita buteae* Ahmad & Shujauddin, 2004, from India

*Hartemita chapini* (Mao, 1945), from Philippines and Malaysia

*Hartemita chinensis* Chen, He & Ma, 1998, from China

*Hartemita coffeana* sp. n., from Vietnam

*Hartemita daklaka* sp. n., from Vietnam

*Hartemita excavata* Chen, He & Ma, 1998, from China and Vietnam

*Hartemita flava* Chen, He & Ma, 1998, from China

*Hartemita khuatbaolinhae* sp. n., from Vietnam

*Hartemita latipes* Cameron, 1910, from Indonesia, East and West Malaysia

*Hartemita maculata* sp. n., from Vietnam, China and Nepal

*Hartemita muirii* (Fullaway, 1919), from Philippines and Japan

*Hartemita nigrotestacea* Belokobylskij & Ku, 2000, from Japan and South Korea

*Hartemita punctata* Chen, He & Ma, 1998, from China

*Hartemita rhadinotarsa* Dangerfield & Austin, 1990, from India, Indonesia and Nepal

*Hartemita rudis* (Mao, 1945), from Philippines

*Hartemita similis* sp. n., from Vietnam

*Hartemita singaporensis* (Mao, 1945), from Singapore, Laos, West and East Malaysia and Vietnam

*Hartemita spasskensis* Belokobylskij, 2005, from Far East Russia

*Hartemita townesi* Dangerfield & Austin, 1990, from China (Taiwan)

*Hartemita vietnamica* sp. n., from Vietnam

##### Key to species of the genus *Hartemita* Cameron

**Table:** 

1	Maximum width of hind basitarsus 1.2–1.6 times apical width of hind tibia and dorsally convex (Figs 5, 28, 46, 64, 67, 71, 86); but sometimes weakly so (Figs 80, 83); if 1.2 times then hind basitarsus 3.8–6.0 times as wide as second hind tarsal segment (Figs 64, 67, 80, 83)	2
–	Maximum width of hind basitarsus 0.8–1.1 times apical width of hind tibia and dorsally nearly straight (Figs 22, 52, 55, 58, 61, 77, 89, 92), but rarely slightly convex (Fig. 16); if 1.1 times then hind basitarsus 2.8–3.5 times as wide as second hind tarsal segment (Figs 40, 49, 74)	11
2	Dorso-apically hind basitarsus strongly protruding, beyond apex of second tarsal segment (Fig. 71); scutellum distinctly convex; maxillary palp 0.7–0.8 times as long as height of head; [head weakly excavate medio-posteriorly in dorsal view; hind tibial spurs yellowish-brown]; Singapore, East and West Malaysia, Laos, *Vietnam	*Hartemita singaporensis* (Mao, 1945)
–	Dorso-apically hind basitarsus weakly or not protruding, not surpassing middle of second tarsal segment (Figs 5, 28, 46, 64, 67, 86); scutellum slightly convex or flat; maxillary palp 1.0–1.5 times as long as height of head	3
3	Ventral margin of clypeus curved medially (Figs 4, 23, 81, 84); hind basitarsus comparatively wide apically (Figs 5, 28, 83), but in *Hartemita chinensis* less so (Fig. 86)	4
–	Ventral margin of clypeus more or less concave or straight medially (Figs 41, 62, 78); hind basitarsus comparatively wide apically (Figs 46, 64, 67, 80)	8
4	Maxillary palp about 1.3 times as long as height of head; hind basitarsus 2.3–2.5 times as long as wide, 4.4–5.0 times as wide as second tarsal segment and 1.4–1.5 times as long as width of apex of hind tibia (Figs 5, 28, 83); propleuron entirely yellow; hind coxa with one black spot	5
–	Maxillary palp about as long as height of head; hind basitarsus 2.8–3.0 times as long as wide, 3.6 times as wide as second tarsal segment and about 1.2 times as long as width of apex of hind tibia (Fig. 86); propleuron with a blackish spot posteriorly; hind coxa with two black spots; [mesosternum black; third hind tarsal segment of female 1.2–1.3 times longer than wide]; Oriental China	*Hartemita chinensis* Chen, He & Ma, 1998
5	Hind basitarsus about 2.3 times as long as remaining tarsal segments and 1.2 times as wide as apex of hind tibia (Fig. 83); third hind tarsal segment of female slightly longer than wide; mesosternum yellow; scutellar sulcus with 3 carinae; hind tarsal claws with 3–4 teeth; Oriental China	*Hartemita flava* Chen, He & Ma, 1998
–	Hind basitarsus about 1.8 times as long as remaining tarsal segments and 1.3–1.4 times as wide as apex of hind tibia (Figs 5, 28); third hind tarsal segment of female about twice as long as wide; mesosternum black; scutellar sulcus with 5–6 carinae; hind tarsal claws with 5–8 teeth	6
7	Hind tarsal claws with 8 large teeth (Fig. 8); face finely punctate; hind basitarsus largely blackish (Fig. 5); temples parallel-sided (Fig. 7) and slightly punctate; Philippines, Japan	*Hartemita muirii* (Fullaway, 1919)
–	Hind tarsal claws with 5 large teeth (Fig. 27); face rugose-punctate; hind basitarsus only apically blackish (Fig. 28); temples rather bulging (Fig. 23), roughly punctate dorsally and rugose-punctate ventrally; Vietnam	*Hartemita khuatbaolinhae* sp. n.
8	Second-fourth hind tarsal segments slender, distinctly longer than wide (Fig. 64); dorsal side of hind basitarsus distinctly more curved than nearly straight ventral side and with a rather distinct apical prominence (Fig. 64); hind femur yellow; [posterior half of notauli widely crenulate; frons entirely smooth]; Oriental China, Nepal	*Hartemita townesi* Dangerfield & Austin, 1990
–	Second-fourth hind tarsal segments robust, slightly longer than wide (Figs 46, 67, 80); dorsal side of hind basitarsus similar to ventral side and truncate apically, without apical prominence (Figs 46, 67, 80); hind femur largely dark brown or black dorsally	9
9	Hind basitarsus elliptical (Fig. 67); face distinctly punctate; mesopleuron below precoxal sulcus coarsely punctate; basal and apical quarter of hind tibia and largely spurs dark brown (Fig. 67); Brunei, *East Malaysia	*Hartemita bruneiensis* Dangerfield & Austin, 1990
–	Hind basitarsus nearly parallel-sided (Figs 46, 80); face punctulate or sparsely punctate; mesopleuron below precoxal sulcus rugulose-punctulate or smooth; hind tibia mainly and spurs yellowish-brown (Figs 46, 80)	10
10	Head in dorsal view comparatively short and occiput weakly concave (Fig. 79); pronotum coarsely rugose-striate; POL 1.2–1.3 times diameter of posterior ocellus; dark part of hind basitarsus close to base of basitarsus (Fig. 80); mesopleuron below precoxal sulcus densely rugulose-punctulate; Japan, South Korea	*Hartemita nigrotestacea* Belokobylskij & Ku, 2001
–	Head in dorsal view comparatively long and occiput distinctly concave (Fig. 42); pronotum largely smooth except for median groove; POL 1.6 times diameter of posterior ocellus; dark part of hind basitarsus distinctly removed from base of basitarsus (Fig. 46); mesopleuron below precoxal sulcus smooth; Vietnam	*Hartemita vietnamica* sp. n.
11	Ventral margin of clypeus more or less curved medio-ventrally (Figs 75, 90) and hind basitarsus 0.8 times wider than apex of hind tibia (Figs 77, 92) and 2.2–2.3 times wider than second hind tarsal segment	12
–	Hind basitarsus 0.9–1.1 times wider than apex of hind tibia (Figs 16, 22, 40, 49, 52, 55, 58, 61, 74, 89) and 2.5–4.0 times wider than second hind tarsal segment, if rarely 0.8 times wider than apex of hind tibia then usually ventral margin of clypeus straight medio-ventrally or nearly so (Fig. 50)	13
12	Hind basitarsus about 1.2 times as long as remaining tarsal segments (Fig. 92); hind basitarsus of male about 5 times as long as wide; face distinctly punctate; fourth hind tarsal segment of male distinctly longer than wide (Fig. 92; female unknown); [maxillary palp about as long as height of head]; Oriental China	*Hartemita punctata* Chen, He & Ma, 1998
–	Hind basitarsus about 1.5 times as long as remaining tarsal segments (Fig. 77); hind basitarsus of male about 4 times as long as wide; face faint transverse rugae; fourth hind tarsal segment of male slightly wider than long (Fig. 77; female unknown); India	*Hartemita buteae* Ahmad & Shujauddin, 2004
13	Hind basitarsus comparatively wide and its apical half largely yellowish (Fig. 16); hind basitarsus 4.0 times as wide as second hind tarsal segment (Fig. 16); occiput deeply concave (Fig. 13); [scutellar sulcus with 5–6 carinae; hind tarsal claws with 5–6 teeth]; Vietnam	*Hartemita coffeana* sp. n.
–	Hind basitarsus comparatively narrow elliptical and its apical half more or less blackish or dark brown (Figs 22, 40, 49, 52, 58, 61, 74), but yellowish-brown in *Hartemita basilaris* (Fig. 55); hind basitarsus 2.8–3.3 times as wide as second hind tarsal segment (Figs 22, 40, 49, 52, 58, 61, 74, 89); occiput slightly to moderately concave (Figs 18, 36, 48, 51, 57, 60, 73), but deeply so in *Hartemita excavata* (Fig. 88)	14
14	Ventral margin of clypeus weakly but evenly curved medially (Figs 53, 56, 59); temple largely smooth, but coarsely punctate in *Hartemita basilaris*; hind basitarsus distinctly narrowed apically (Figs 55, 58), but slightly so in *Hartemita chapini* (Fig. 61); length of body 6–9 mm, but in *Hartemita chapini* up to 5 mm	15
–	Ventral margin of clypeus nearly straight to slightly concave medially (Figs 17, 35, 47, 50; 72, 87); temple more or less punctate; hind basitarsus slightly narrowed apically (Figs 22, 40, 49, 52, 74, 89); length of body 4.0–6.3 mm	18
15	Head coarsely punctate (Fig. 53); hind basitarsus largely yellowish-brown; dorsal margin of clypeus evenly curved (Fig. 53); second-fifth hind tarsal segments less slender (Fig. 55); vein SR1 of fore wing almost vertical basally; [hind coxa with two black spots dorsally]; Indonesia, *East Malaysia	*Hartemita basilaris* Dangerfield & Austin, 1990
–	Head smooth or mainly finely punctate (Figs 56, 59); hind basitarsus largely dark brown or blackish; dorsal margin of clypeus straight or slightly sinuate (Figs 56, 59); second-fifth hind tarsal segments slender (Figs 58, 61); vein SR1 of fore wing distinctly oblique basally	16
16	Outer side of hind tibia partly dark brown apically (Fig. 61); second submarginal cell of fore wing 3.3–4.0 times as long as wide near level of vein r; second-fifth hind tarsal segment less robust (Fig. 61); anterior transverse carina of propodeum absent; length of body 5.0–6.1 mm]; Philippines	*Hartemita chapini* (Mao, 1945)
–	Outer side of hind tibia (except for dark brown basal ring) yellowish apically (Figs 96, 100); second submarginal cell of fore wing 3.6–4.6 times as long as wide near level of vein r; anterior transverse carina of propodeum more or less developed; length of body 6–9 mm	17
17	Mesoscutum completely black near notauli (Fig. 95); OOL black (Fig. 93); ovipositor sheath dark brown; anterior transverse carina of propodeum moderately to weakly developed Indonesia, East and West Malaysia	*Hartemita latipes* Cameron, 1910
–	Mesoscutum brownish-yellow near notauli (Fig. 99); OOL yellow (Fig. 97); ovipositor sheath yellowish-brown; anterior transverse carina of propodeum coarsely developed; [apical rim of clypeus in Chinese specimens brownish-yellow]; Oriental China, Vietnam, Nepal	*Hartemita maculata* sp. n.
18	Hind basitarsus 2.0–2.3 times as long as remainder of tarsus (Figs 49, 74); inner hind spur 0.5–0.6 times as long as hind basitarsus; maxillary palp about 1.3 times as long as height of head	19
–	Hind basitarsus 1.4–1.8 times as long as remainder of tarsus (Figs 22, 40, 52, 89); inner hind spur 0.7 times as long as hind basitarsus; maxillary palp about as long as height of head or slightly shorter, but 1.3–1.4 times as long in *Hartemita rhadinotarsa* and *Hartemita similis*	20
19	Clypeus concave medio-ventrally (Fig. 47); second submarginal cell of fore wing about 4 times as long as wide; medio-posteriorly mesoscutum with a wide depressed area; Philippines	*Hartemita rudis* (Mao, 1945)
–	Clypeus truncate and protruding medio-ventrally (Fig. 72); second submarginal cell of fore wing 3.3–3.4 times as long as wide; mesoscutum medio-posteriorly without a wide depressed area; Far East Russia	*Hartemita spasskensis* Belokobylskij, 2005
20	Tarsal claws with 5–6 teeth; hind tibia yellow apically; [second and third metasomal tergites with black spots laterally; second submarginal cell of fore wing 3.0–3.3 times as long as wide]; Oriental China	*Hartemita excavata* Chen, He & Ma, 1998
–	Tarsal claws with 2–4 teeth (Figs 21, 39); hind tibia dark brown apically (Figs 22, 40, 52)	21
21	Second and third metasomal tergites black laterally; vein 3-SR of fore wing 2.3–2.4 times as long as vein 2-SR and 0.7 times as long as vein SR1 (Fig. 37); Vietnam	*Hartemita similis* sp. n.
–	Second and third tergites brownish-yellow laterally; vein 3-SR of fore wing 1.5–1.7 times as long as vein 2-SR and 0.5–0.6 times as long as vein SR1 (Fig. 19)	22
22	Apical 0.3–0.4 of hind tibia dark brown or blackish (Fig. 52); vein 1-SR of fore wing gradually merging into vein 1-M; mesoscutum with 3 blackish patches; India, Nepal, Indonesia	*Hartemita rhadinotarsa* Dangerfield & Austin, 1990
–	Apical 0.2 of hind tibia dark brown or blackish (Fig. 22); vein 1-SR of fore wing angled with vein 1-M (Fig. 19); mesoscutum brownish-yellow, without blackish patches; Vietnam	*Hartemita daklaka* sp. n.

## Descriptions

### Hartemita coffeana sp. n.

urn:lsid:zoobank.org:act:84E385BC-439E-4A08-A798-61E3B7C464E7

http://species-id.net/wiki/Hartemita_coffeana

Figs 12–16

#### Type material.

Holotype, female (IEBR), “Card.059”, “[S Vietnam:] Dak Lak, Easo, coffee farm, MT, 108°37’E, 02.vii.2008, Ngo Hien”.

#### Diagnosis.

The new species is close to *Hartemita rhadinotarsa* Dangerfield & Austin, but differs by having epistomal suture indistinctly developed, with the rugosities of the face and the punctures of the clypeus distinct (suture distinct and face and clypeus finely punctate in *Hartemita rhadinotarsa*); the occiput deeply concave (moderately concave in *Hartemita rhadinotarsa*); the hind tarsal segments (excerpt basitarsus) 0.6 times as long as hind basitarsus (0.8 times in *Hartemita rhadinotarsa*) and the hind tarsal claws with 5 teeth (2–4 teeth in *Hartemita rhadinotarsa*). Differs from *Hartemita excavata* Chen, He & Ma by having the transverse diameter of the eye in dorsal view 1.3 times as long as the temple (0.9 times in *Hartemita excavata*), POL 1.5 times OD (1.3 times in *Hartemita excavata*) and the scutellar sulcus with 5 cross-carinae (3 cross-carinae in *Hartemita excavata*).

#### Description.

Holotype, female, body length 6.2 mm, fore wing length 7.3 mm, antenna 7.8 mm.

*Head*. Antennal segments 52; third segment 1.2 times as long as fourth segment; length of third, fourth and penultimate segments 2.3, 1.9 and 2.0 times their width, respectively; head width 2.2 times its median length; occiput deeply excavate (Fig. 13); temple behind eyes convex anteriorly, gradually narrowed posteriorly (Fig. 13); length of temple 0.9 times transverse diameter of eye; OOL:POL:OD = 19:9:6; frons wide and with a median carina (Fig. 13); eye glabrous, width of face 1.5 times height of eye; clypeal margin nearly straight medially, epistomal suture indistinct; malar space 1.4 times width of mandible; face largely rugose; clypeus shiny and punctate; temple very shiny and with sparse but large and discrete punctures, distance between punctures twice diameter of puncture; frons smooth laterally, striate medially and transversely rugose posteriorly.

*Mesosoma*. Length of mesosoma 1.1 times its height; pronotal trough sparsely crenulate medially, remainder of pronotal side sparsely punctate; propleuron sparsely punctate; notauli flattened posteriorly, narrowed anteriorly and crenulate; scutellar sulcus with 5 cross-carinae; scutellum convex, punctate; median arch of metanotum with lateral cross-carinae (Fig. 15); mesopleuron shiny, medially with sparse punctures and rugose-punctate anteriorly; precoxal sulcus crenulate anteriorly and rugose posteriorly; mesosternum areolate-punctate; metapleuron and propodeum rugose.

*Wings*. Length of fore wing 2.9 times its maximum width; length of pterostigma 4.0 times its median width; r:2-SR:3-SR = 18:20:40; second submarginal cell of fore wing 3.4 times longer than its maximum width (Fig. 14); vein 1-CU1 0.4 times as long as vein 2-CU1; vein 3-SR joining SR1 at 100°. Length of hind wing 4.3 times its width; vein M+CU 0.4 times as long as vein 1-M.

*Legs*. Length of hind femur 4.2 times its width; length of hind tibia 4.4 times its apical width; hind basitarsus slightly produced apically (Fig. 16), flattened, not broadly laminate, 2.8 times longer than wide and as wide as apical width of hind tibia (Fig. 16);second-fifthhind tarsal segments 0.6 times as long as hind basitarsus; inner hind tibial spur 0.6 times as long as hind basitarsus; hind tarsal claw with 5 teeth; hind coxa and outer side of hind femur rugose-punctate; upper side of hind tibia with some spines.

*Metasoma*. Second metasomal tergite 0.8 times as long as third tergite; ovipositor sheath short; ovipositor curved.

*Colour*. Body yellow; scapus yellow, black apically and laterally; frons black medially, yellow laterally; stemmaticum and vertex black; temple black along occiput margin; hind femur yellow with black band on upper side; hind tibia yellow, black basally and apically; hind basitarsus yellow, black apically; hind spurs and tarsus (except basitarsus) dark brown.

*Male*. Unknown.

#### Distribution.

S Vietnam: Dak Lak.

#### Etymology.

After the genus *Coffea* Linnaeus, because the new species was collected at a coffee farm.

**Figures 1–11.**
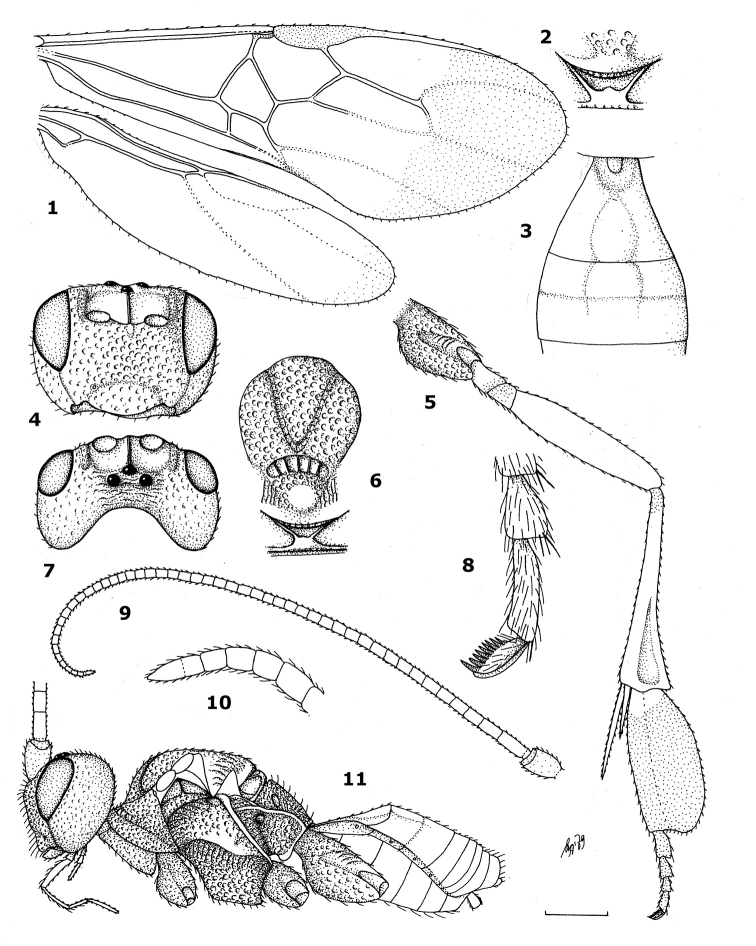
*Hartemita muirii* (Fullaway), female, holotype. **1** wings **2** metanotum dorsal **3** first-third metasomal tergites dorsal **4** head frontal **5** hind leg **6** mesosoma dorsal **7** head dorsal **8** outer hind claw **9** antenna **10** apex of antenna **11** habitus lateral. 1, 5, 9, 11: 1.0 × scale bar 2: 2.6 × 3, 4, 6, 7: 1.3 × 8, 10: 5.0 ×.

**Figures 12–16. F2:**
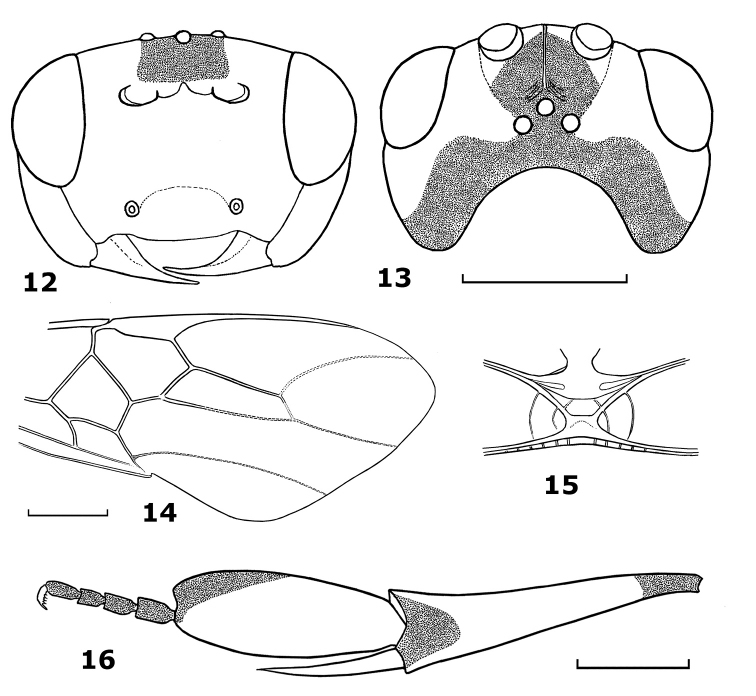
*Hartemita coffeana* sp. n., female, holotype. **12** head frontal **13** head dorsal **14** fore wing **15** metanotum dorsal **16** hind tibia and tarsus.

### Hartemita daklaka sp. n.

urn:lsid:zoobank.org:act:EA72044F-96F5-4DEA-994F-BB73E93D9CB8

http://species-id.net/wiki/Hartemita_daklaka

Figs 17–22

#### Type material.

Holotype, male (IEBR), “Card.058”, “[S Vietnam:] Dak Lak, Easo, coffee farm, MT, 108°37’E, 02.vii.2008, Ngo Hien”.

#### Diagnosis.

Occiput moderately concave; medio-ventral margin of clypeus slightly concave; mesopleuron entirely smooth; precoxal sulcus crenulate anteriorly and smooth posteriorly; hind tarsal claw with 3–4 teeth; hind basitarsus as wide as apical part of hind tibia, parallel-sided, flattened and not broadly laminate or produced apically.

#### Description.

Holotype, male, body length 4.9 mm, fore wing length 5.1 mm, antenna 6.5 mm.

**Figures 17–22. F3:**
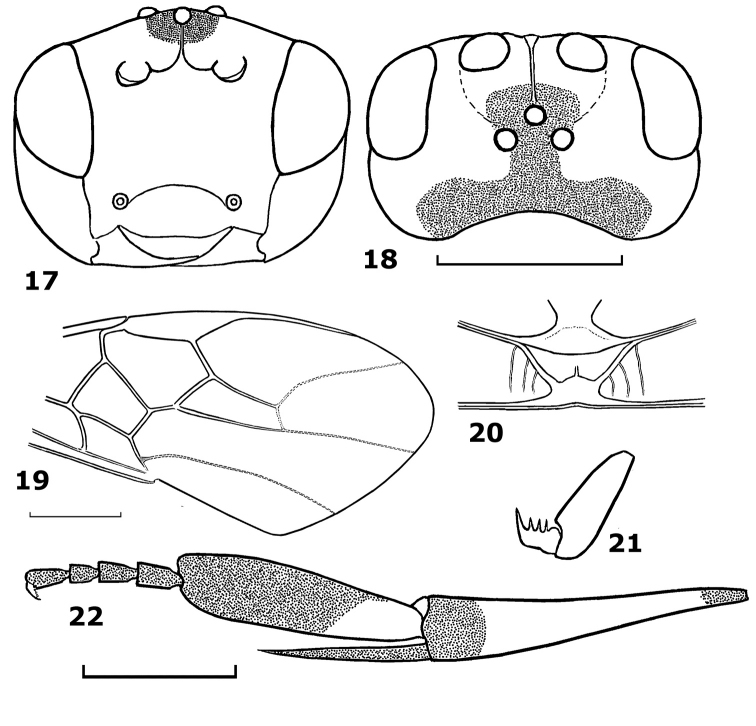
*Hartemita daklaka* sp. n., male, holotype. **17** head frontal **18** head dorsal **19** fore wing **20** metanotum dorsal **21** hind tarsal claw **22** hind tibia and tarsus.

*Head*. Antennal segments 43; third segment 1.2 times as long as fourth segment; length of third, fourth and penultimate segments 2.2, 1.8 and 1.0 times their width, respectively; epistomal suture distinct and evenly curved (Fig. 17); clypeal margin slightly concave medially (Fig. 17); in dorsal view head width 1.8 times its median length; occiput moderately concave (Fig. 18); temple behind eyes convex anteriorly, roundly narrowed posteriorly (Fig. 18); length of temple 0.65 times transverse diameter of eye; OOL:POL:OD= 13:7:5; frons deep; eye glabrous, transverse diameter of eye 1.8 times its width dorsally; width of face 1.4 times height of eye; malar space 1.9 basal width of mandible (Fig. 17); face shiny and largely punctate laterally, face medially and clypeus sparsely finely punctate; area around facial node rugose.

*Mesosoma*. Length of mesosoma 1.1 times its height; pronotal trough crenulate medially, remainder of pronotal side finely punctate; notauli shallow and rugose posteriorly; scutellar sulcus with 5 cross-carinae (in paratype 3); scutellum convex and largely punctate; propleuron shiny and with sparse fine punctures; mesopleuron shiny and largely smooth medially; precoxal sulcus and mesosternum areolate-punctate; median arch of metanotum without lateral cross-carinae (Fig. 20); metapleuron and propodeum dull and rugose.

*Wings*. Length of fore wing 2.6 times its maximum width; pterostigma medium-sized; length of pterostigma 3.8 its median width; r:2-SR:3-SR = 9:16:21; length of second submarginal cell of fore wing 3.3 times its maximum width; vein 1-CU1 0.14 times vein 2-CU1; vein 3-SR joining SR1 at 100° (Fig. 19). Length of hind wing 4.0 times its width; vein M+CU 0.4 times as long as vein 1-M.

*Legs*. Length of hind femur 4.6 times its width; length of hind tibia 5.3 times its apical width; hind basitarsus flattened, not broadly laminate and not produced apically (Fig. 22), 4.0 times as long as wide; hind basitarsus as wide as apical width of hind tibia; second-fifth hind tarsal segments comparatively long (Fig. 22), 0.6 times as long as hind basitarsus; inner hind tibial spur 0.7 times as long as hind basitarsus; hind tarsal claw with 3 teeth (Fig. 21).

*Metasoma*. Metasoma 0.9 times as long as mesosoma; second metasomal tergite as long as third tergite or slightly longer; ovipositor sheath very short; ovipositor curved.

*Colour*. Body yellow; antenna dark brown; scapus black, but yellow ventrally; palpi brown, except first yellow segment; frons black posteriorly and yellow anteriorly (Fig. 18); vertex black; middle trochantellus, basal ring of middle tibia, middle spurs and tarsus (except yellow base of basitarsus) dark brown; hind femur yellow, but dark brown dorsally; hind tibia yellow, black basally and apically; hind basitarsus black, but yellow basally; hind trochanter and trochantellus, spurs and tarsus dark brown; wings brown, smoky apically.

*Female*. Unknown.

#### Distribution.

S Vietnam: Dak Lak.

#### Etymology.

Named after the province of its type locality: Dak Lak.

### Hartemita khuatbaolinhae sp. n.

urn:lsid:zoobank.org:act:C26438F4-7228-40B7-BD5C-09B1C1653211

http://species-id.net/wiki/Hartemita_khuatbaolinhae

Figs 23–28

#### Type material.

Holotype, female (IEBR), “Card.001”, “[NE Vietnam:] Phu Tho, Xuan Son NP, forest, 10.v.2005, P. Th. Nhi.” Paratypes: 1 female (RMNH), “Card.002”, data as holotype; 1 female (IEBR), “Card.010”, “[CN Vietnam:] Ha Tinh, Huong Son, Son Hong, 23.iv.2004, Tr. X. Lam”.

#### Diagnosis.

The new species is similar to *Hartemita muirii* (Fullaway), but differs by having the face rugose-punctate (punctate in *Hartemita muirii*; Fig. 4), temples bulging (subparallel-sided in *Hartemita muirii*; Fig. 7), hind basitarsus largely yellow (largely blackish in *Hartemita muirii*; Fig. 5) and the hind claw with 5 teeth (8 teeth in *Hartemita muirii*; Fig. 8).

#### Description.

Holotype, female, body length 7.7 mm, fore wing length 8.2 mm, antenna 9.5 mm.

**Figures 23–28. F4:**
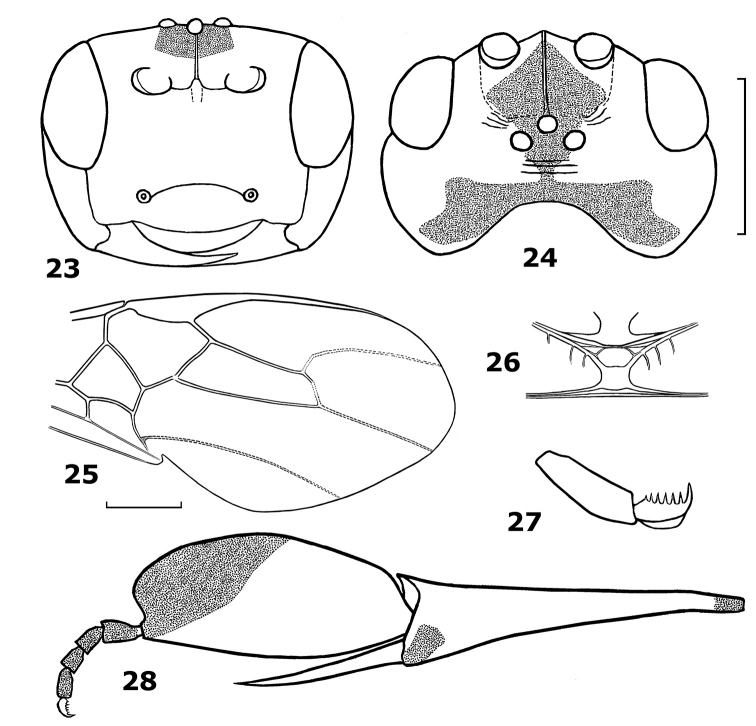
*Hartemita khuatbaolinhae* sp. n., female, holotype. **23** head frontal **24** head dorsal **25** fore wing **26** metanotum dorsal **27** hind tarsal claw **28** hind tibia and tarsus.

*Head*. Antennal segments 52 (paratype: 51); third antennal segment 1.3 times fourth segment; length of third, fourth and penultimate segments 2.1, 1.6 and 1.6 times their width, respectively; eye glabrous, twice as long as its lateral width; width of face 1.5 times as long as height of eye; clypeal margin convex medio-ventrally (Fig. 23); epistomal suture distinct and evenly curved; malar space 1.2 times basal width of mandible (Fig. 23); in dorsal view head transverse, its width nearly twice as long as its median length; occiput deeply excavate (Fig. 24); temple behind eyes convex anteriorly and roundly narrowed posteriorly; length of temple 1.2 times transverse diameter of eye (Fig. 24); width of eye 0.55 times temple laterally; OOL:POL:OD= 15:9:6; face rugose-punctate; clypeus largely punctate; frons smooth and with a median carina; area around ocelli with transverse and dense rugae; temple largely rugose ventrally and with large punctures dorsally.

*Mesosoma*. Length of mesosoma 1.3 times its height; pronotal trough rugose dorsally, remainder of pronotal side smooth; notauli deep and areolate posteriorly; scutellar sulcus with 6 cross-carinae (paratype with 5); scutellum convex and punctate; mesoscutum rugose-punctate; median arch of metanotum with short lateral cross-carinae (Fig. 26); mesopleuron smooth medially and rugose-punctate anteriorly; precoxal sulcus shallow; mesosternum rugose-punctate; metapleuron and propodeum rugose.

*Wings*. Length of fore wing 2.3 times its maximum width; length of pterostigma 4.3 times its median width; r:2-SR:3-SR = 16:26:53; length of second submarginal cell of fore wing 3.2 times its maximum width; vein 1-SR+M slightly sinuate (Fig. 25); vein 1-CU1 0.5 times vein 2-CU1 (10:22); vein 3-SR joining SR1 at 90°. Length of hind wing 4.6 times its width; vein M+CU 0.3 times as long as vein 1-M.

*Legs*. Length of hind femur 4.25 times its median width; hind basitarsus broadly laminate, slightly produced apically and 2.1 times as long as wide (Fig. 28); width of hind basitarsus 1.4 times apical width of hind tibia; second-fifth hind tarsal segments 0.6 times as long as hind basitarsus (Fig. 28); inner hind tibial spur 0.6 times as long as hind basitarsus; hind claw with 5 teeth (Fig. 27).

*Metasoma*. Metasoma as long as mesosoma; second metasomal tergite 0.85 times as long as third tergite; ovipositor sheath very short, round apically; ovipositor almost straight.

*Colour*. Body yellow; antenna dark brown; scapus yellow, but apex and outer side dark brown; frons and stemmaticum black; vertex yellow anteriorly and black posteriorly; temple partly black dorsally; median and lateral lobes of mesoscutum and mesosternum black; middle trochantellus, apical upper and lower sides of hind coxa, hind trochanter apically, hind trochantellus, apical third of basitarsus and hind tarsus (except basitarsus) black; fore wing brown, but smoky apically.

*Male*. Unknown.

#### Distribution.

N Vietnam: Phu Tho and C Vietnam: Ha Tinh.

#### Etymology.

The species named after the granddaughter of the first author, Khuat Bao Linh.

### Hartemita maculata sp. n.

urn:lsid:zoobank.org:act:46FBE7D8-FA4A-45CC-8EF7-3D619335097

http://species-id.net/wiki/Hartemita_maculata

Figs 29–34 91 94

#### Type material.

Holotype, female (IEBR), “Card.026”, “[C.N.Vietnam:] Nghe An, Con Cuong, Pu Mat NP, 250 m, 12.ix.2005, P. Th. Nhi”. Paratypes: 2 males (IEBR, RMNH), “Card.052”, “Card.053”, “[N.E. Vietnam:] Thai Nguyen, Dai Tu, Minh Tien, MT, 21°43’N 105°34’E, 350 m, 10–15.ix.2007, K. D. Long”; 1 female (IEBR), “Card.054”, id., but tea farm, 31.v.2008, K. D. Long; 1 female (RMNH), “Card.066”, “[N.E. Vietnam:] Vinh Phuc,Tam DaoNP, 100 m, MT, 30.iv.2008, P. H. Thai”; 1 male (IEBR), “Card.032”, “[C. Vietnam:] Thua Thien-Hue, Nam Dong, MT, 2–6.v.2005, N. Q. Truong”; 2 females (IEBR, RMNH), “Card.067”, “Card.068”, 6 males (IEBR, RMNH), “Card.069”, “Card.070”, “[N.E. Vietnam:] Phu Tho (Xuan Son NP), garden, MT, 20–25.v.2010, K.D. Long, N. H. Thao”; “Card.071”, “Card.072”, id., but 19–21.vi.2009; “Card.073”, id., but 29.vi-05.vii.2009; “Card.074”, id. but 25–30.vii.2009; 1 female (RMNH) “N. Vietnam: Ninh Binh, Cuc Phuong N.P., nr entrance, [Mal. trap], c 225 m, 14.iv.-1.v.2000, Mai Phu Quy, RMNH’00”; 1 female (RMNH) “[China:], Hunan, Lianyun Mt., cotton-shelter, 2.vii.2007, 28°30.203’N 113°48.619’E, altitude 590 m, Li Ze-jan”; 1 male (RMNH), [China:] Hunan, Mufu Mt., Yanziping, altitude 1330 m, 29.vi. 2007, 28°58.524’N 113°49.638’E, Li Ze-jan”; 1 female (RMNH), “China: Fujian, Nanjin, 30.v.1991, no. 96 9320, Liu Changmin, RMNH’99”.

#### Diagnosis.

The new species is similar to *Hartemita latipes* Cameron, but differs by having the mesoscutum brownish-yellow near the notauli (Fig. 93) (completely black near the notauli in *Hartemita latipes*; Fig. 89); OOL yellow (Fig. 91) (black in *Hartemita latipes*; Fig. 87); the ovipositor sheath yellowish-brown (dark brown in *Hartemita latipes*); the anterior transverse carina of the propodeum coarsely developed (moderately to weakly developed in *Hartemita latipes*); the mesosternum and the mesopleuron of female yellowish-brown (largely black in *Hartemita latipes*) and the tarsal claws with 4–5 large teeth (Fig. 32) and 0–2 small teeth (2–3 (rarely 4) large teeth and 3–4 small teeth in *Hartemita latipes*).

Description. Holotype, female, body length 8.0 mm, fore wing length 7.6 mm, antenna 7.8 mm.

**Figures 29–34. F5:**
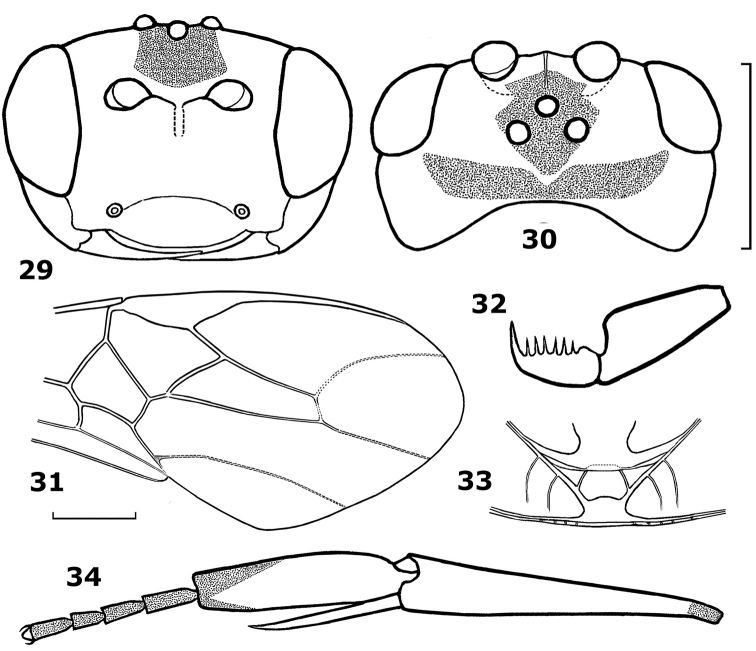
*Hartemita maculata* sp. n., female, holotype. **29** head frontal **30** head dorsal **31** fore wing **32** hind tarsal claw **33** metanotum dorsal **34** hind tibia and tarsus.

*Head*. Antennal segments: 43 (paratypes: 41, 42 (2) or 43); third antennal segment 1.4 times as long as fourth; length of third, fourth and penultimate segments 1.9, 1.6, 2.0 times their width respectively; clypeal margin convex medio-ventrally (Fig. 29); epistomial suture distinct, curved; width of face 1.3 times height of eye; malar space equal to basal width of mandible; in dorsal view head width 2.3 times its median length; occiput weakly concave (fig. 30); temple behind eyes almost perpendicular posteriorly (Figs 30, 97); length of temple 0.9 times as long as transverse diameter of eye; OOL:POL:OD = 16:8:6; frons concave; eye glabrous; laterally length of eye twice its width and 0.75 times temple.

*Mesosoma*. Length of mesosoma 1.3 times its height; pronotal trough shiny and smooth as surroundings; notauli shallow and smooth; scutellar sulcus with 3 cross-carinae; scutellum, mesopleuron and mesosternum shiny and smooth; precoxal sulcus crenulate anteriorly and smooth posteriorly; metapleuron smooth anteriorly and rugose posteriorly; median arch of metanotum with lateral cross-carinae (Fig. 33); propodeum largely rugose.

*Wings*. Length of fore wing 2.7 times its maximum width; pterostigma length 3.2 times its median width; vein r arising near middle of pterostigma; r:2-SR:3-SR = 13:20:35; length of second submarginal cell of fore wing 3.5 times its width; vein 1-CU1 0.4 times vein 2-CU1; vein 3-SR joining vein SR1 at 100° (Fig. 31). Length of hind wing 4.3 times its width; vein M+CU 0.3 times vein 1-M.

*Legs*. Length of hind femur 4.2 times its width; hind basitarsus flattened, not broadly laminate and not produced apically, its width 0.9 times distal width of hind tibia; length of hind basitarsus 3.75 times as long as its width (Figs 34, 100); hind tarsal segments 2–5 not shortened, 0.8 times as long as hind basitarsus (Fig. 100); inner hind tibial spur 0.7 times as long as hind basitarsus; outer side of hind tibia with long sparse spines; hind claw with 5 large teeth and 1 small tooth (Fig. 32).

*Metasoma*. Metasoma 1.2 times length of mesosoma dorsally; second tergite 0.8 times as long as third segment; ovipositor sheath very short, round apically; ovipositor curved.

*Colour*. Body yellow; antenna brown; scapus dark brown laterally; frons, stemmaticum black; vertex black posteriorly; lateral and middle lobes of mesoscutum, mesosternum black; wings brown, but parastigma yellow; apex of fore wing (behind vein r-m) darker; near apex of hind coxa with a large black spot; trochantellus, basal ring of hind tibia, apex of hind basitarsus and remainder of hind tarsus dark brown; basal corner of second metasomal tergite and fourth and fifth tergites black apically.

*Male*. Body length 7.0–7.8 mm, fore wing length 6.8–8.3 mm, antenna 7.8–8.4 mm; antennal segments 40–44.

#### Distribution.

N Vietnam: Ninh Binh, Vinh Phuc, Thai Nguyen and Phu Tho and C Vietnam: Nghe An and Thua Thien-Hue; Oriental China and Nepal.

#### Etymology.

The species is named **“***maculata***”,** because of the distinctly maculate mesoscutum.

#### Notes.

Specimens from Vietnam have the hind claws with 5–6 teeth and the propodeum rugose with a faint transverse carina anteriorly. Most common species in North and Central Vietnam. Runs in the key by Dangerfield and Austin (1990) to *Hartemita latipes* Cameron, but this species has a Sundaland distribution and differs as indicated above. The only specimen reported as *Hartemita chapini* (Mao) from Malaysia has been examined and belongs to *Hartemita latipes*. As a result, *Hartemita chapini* is a species only known from the Philippines.

### Hartemita similis sp. n.

urn:lsid:zoobank.org:act:1B96DB85-C911-40BE-ACF2-A9A7E3BD0296

http://species-id.net/wiki/Hartemita_similis

Figs 35–40

#### Type material.

Holotype, male (IEBR), “Card.057”, “[S Vietnam:] Dak Lak, Easo, coffee farm, MT, [ca 12°45’N] 108°37’E, 02.vii.2008, Ngo Hien”. Paratype: 1 male (RMNH), “S. Vietnam: Dong Nai, Cat Tien N.P., Bird trail, Mal. trap 9–12, c 100 m, 1–9.x.2005, C. v. Achterberg & R. de Vries, RMNH’05”.

#### Diagnosis.

The new species is similar to *Hartemita punctata* Chen, He & Ma, but differs by having the ventral clypeal margin moderately concave medially (nearly straight in *Hartemita punctata*; Fig. 90), the malar space 1.2 times as long as the basal width of the mandible (equal in *Hartemita punctata*), hind tibia dark brown apically (yellow in *Hartemita punctata*; Fig. 92) and the basitarsus 3.7 times its median width (5.0 times in *Hartemita punctata*; Fig. 92).

#### Description.

Holotype, male, body length 5.9 mm, fore wing length 5.6 mm, antenna 6.5 mm.

**Figures 35–40. F6:**
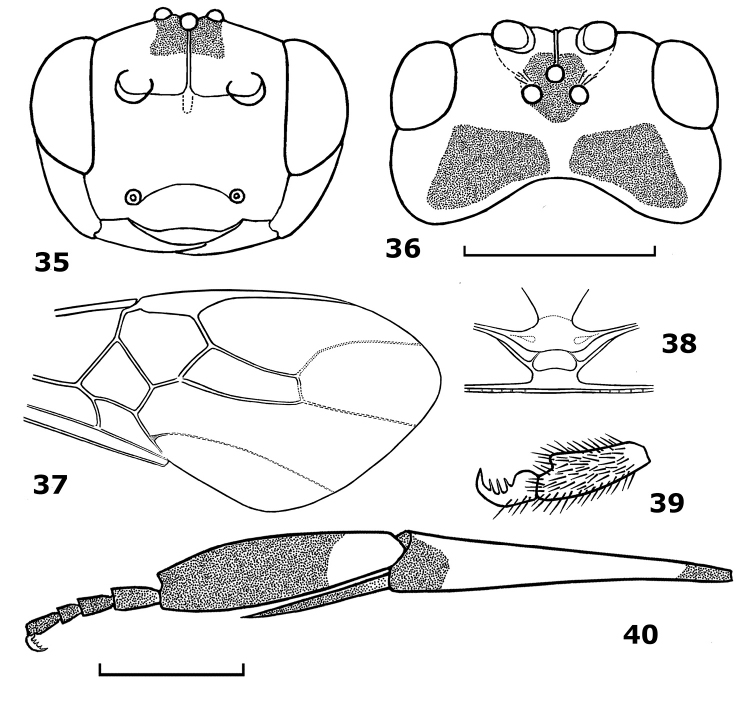
*Hartemita similis* sp. n., male, holotype. **35** head frontal **36** head dorsal **37** fore wing **38** metanotum dorsal **39** hind tarsal claw **40** hind tibia and tarsus.

*Head*. Antennal segments 43; third segment 1.2 times as long as fourth segment; length of third, fourth and penultimate segments 2.0, 1.7 and 1.3 times their width, respectively; eye glabrous, width of face 1.4 times height of eye; clypeal margin moderately concave medially (Fig. 35); epistomal suture distinct and curved; malar space 1.2 times width of mandible (Fig. 35); in dorsal view head width twice its median length; frons narrow; occiput moderately concave (Fig. 36); temple behind eyes convex anteriorly and roundly narrowed posteriorly (Fig. 36); length of temple as long as transverse diameter; OOL:POL:OD = 15:6:5; frons with a median carina (Fig. 35). Face and clypeus shiny and sparsely punctate; vertex and temple shiny and sparsely punctate.

*Mesosoma*. Length of mesosoma 1.2 times its height; pronotal trough crenulate medially, remainder of pronotal side rugose dorsally and smooth ventrally; notauli narrow and more or less flat; scutellar sulcus with 3 cross-carinae; median arch of metanotum with a pair of lateral cross-carinae (Fig. 38); middle and lateral lobes of mesoscutum rugose-punctate; scutellum punctate; mesopleuron smooth medially, rugose dorsally; precoxal sulcus wide; mesosternum areolate-punctate; propodeum rugose.

*Wings*. Length of fore wing 2.5 times its maximum width; length of pterostigma 3.5 times its median width; r:2-SR:3-SR= 8:11:27; length of second submarginal cell 3.7 times its maximum width; vein 1-CU1 0.3 times vein 2-CU1; vein 3-SR joining vein SR1 at 100° (Fig. 37). Length of hind wing 4.7 times its width; vein M+CU 0.5 times as long as vein 1-M.

*Legs*. Length of hind femur 4.3 times its middle width; length of hind tibia 5.1 times its apical width; hind basitarsus flattened, not broadly laminate and not produced apically (Fig. 40); hind basitarsus as wide as apical width of hind tibia and 3.7 times as long as wide; inner hind tibial spur 0.6 times as long as hind basitarsus; second-fifth hind tarsal segments 0.54 times as long as hind basitarsus (Fig. 40); hind tarsal claw with 3 teeth (Fig. 39).

*Metasoma*. Metasoma 1.2 times longer than mesosoma; second metasomal tergite longer than third tergite.

*Colour*. Body yellow; palpi brown; antenna brown, scapus yellow, but outer side dark brown; stemmaticum and vertex black, but separated by yellow area; middle and lateral lobes of mesoscutum and mesosternum black; middle leg yellow, but trochanter apically, trochantellus, tibia basally, spurs and tarsus dark brown (but basitarsus yellow basally); upper apex of hind coxa, trochanter and trochantellus, upper side of hind femur, tibia basally and apically, spurs and hind tarsus dark brown; wing brown and smoky apically; second-third metasomal tergites laterally (but less developed on third tergite) and fourth-seventh tergites medially black.

*Female*. Unknown.

#### Distribution.

S Vietnam: Dak Lak, Dong Nai.

#### Etymology.

Named “*similis*” (Latin for “like”, “resembling”), because it is similar to *Hartemita punctata*.

### Hartemita vietnamica sp. n.

urn:lsid:zoobank.org:act:03B9AA0E-9D47-4649-9447-A651436CD0A7

http://species-id.net/wiki/Hartemita_vietnamica

Figs 41–46

#### Type material.

Holotype, female (IEBR), “Card.065”, “[NE Vietnam:] Vinh Phuc, Tam Dao NP, 100 m, MT, 30.v.2008, P. H. Thai”. Paratype: 1 male (RMNH), “Card.039”, “[CN Vietnam:] Nghe An, Con Cuong, Pu Mat NP, 22.iv.2006, P. Th. Nhi”.

#### Diagnosis.

The new species is similar to *Hartemita bruneiensis* Dangerfield & Austin, but differs by having the occiput deeply excavate (weakly excavate in *Hartemita bruneiensis*; Fig. 66); vein 3-SR joining vein SR1 at 100° (90° in *Hartemita bruneiensis*); hind tibia yellow ventrally (dark brown ventrally; Fig. 67); hind claw with 3 teeth (4–7 teeth in *Hartemita bruneiensis*) and scutellum rugose-punctate (punctate in *Hartemita bruneiensis*).

#### Description.

Holotype, male, body length 6.0 mm, fore wing length 6.8 mm, antenna 7.1 mm.

**Figures 41–46. F7:**
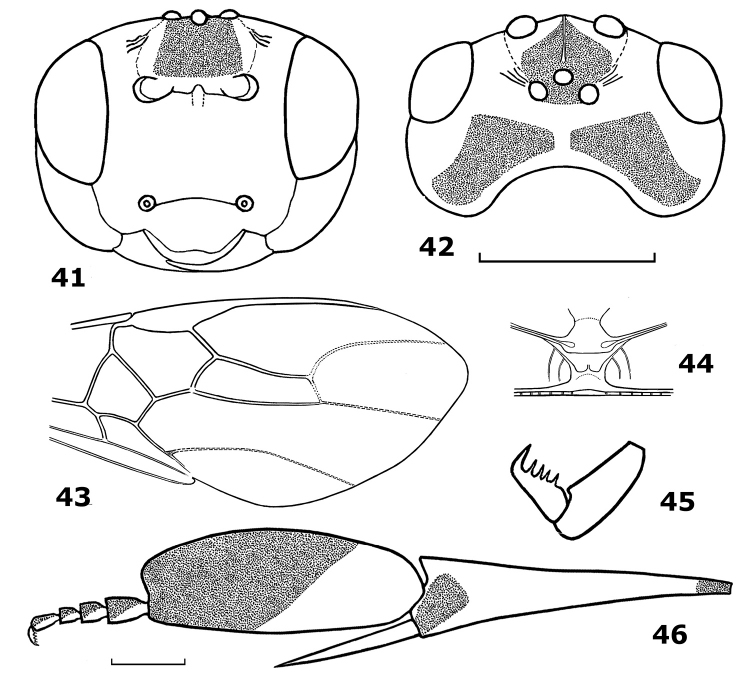
*Hartemita vietnamica* sp. n., male, holotype. **41** head frontal **42** head dorsal **43** fore wing **44** metanotum dorsal **45** hind tarsal claw **46** hind tibia and tarsus.

*Head*. Antennal segments 46 (paratype 44); third segment 1.25 times as long as fourth segment; length of third, fourth and penultimate segments 2.0, 1.6 and 1.0 times their width, respectively; eye glabrous, width of face 1.5 times height of eye; clypeal margin slightly concave medially (Fig. 41); epistomal suture distinct and curved; malar space 1.4 times width of mandible; in dorsal view head twice wider than its median length; occiput deeply concave (Fig. 42); temple behind eyes convex anteriorly and roundly narrowed posteriorly; length of temple nearly as long as transverse diameter of eye; OOL:POL:OD = 15:8:5; frons with a median carina (Fig. 42); face and clypeus sparsely punctate.

*Mesosoma*. Length of mesosoma 1.2 times its height; pronotal trough rugose dorsally, remainder of pronotal side smooth; notauli deep and crenulate anteriorly, nearly separated posteriorly by a carina; scutellar sulcus with 3 cross-carinae; mesoscutum punctate; scutellum rugose-punctate; median arch of metanotum with lateral cross-carinae (Fig. 44); mesopleuron smooth medially; precoxal sulcus shallow; mesosternum areolate-punctate; metapleuron and propodeum rugose.

*Wings*. Length of fore wing 2.7 times its maximum width; pterostigma length 4.0 times its median width; r:2-SR:3-SR = 10:15:35; second submarginal cell of fore wing length 3.4 times its maximum width; vein 1-CU1 0.4 times as long as vein 2-CU1; vein 3-SR joining vein SR1 at 100° (Fig. 43). Length of hind wing 3.9 times its maximum width; vein M+CU 0.4 times as long as vein 1-M.

*Legs*. Length of hind femur 5.2 times its width; hind basitarsus broadly laminate, slightly produced apically; length of hind tibia 3.5 times its apical width; hind basitarsus 2.7 times as long as wide (Fig. 46); hind basitarsus 1.3 times wider than apical width of hind tibia; inner hind tibial spur 0.5 times as long as hind basitarsus; second-fifth hind tarsal segments 0.4 times as long as hind basitarsus (Fig. 46); hind tarsal claw with 4 teeth (Fig. 45).

*Metasoma*. Second tergite shorter than third tergite.

*Colour*. Body and palpi yellow; antenna brown, but scapus yellow with dark brown spot apically and on outer side; frons black; vertex yellow anteriorly and black posteriorly (Fig. 42); middle and lateral lobes of mesoscutum black; mesosternum black dorsally and yellow ventrally; middle leg yellow, but outer side of trochantellus black; hind coxa dorso-apically, trochanter and trochantellus, basal ring of hind tibia, apical half of hind basitarsus dark brown or black; second-fifth hind tarsal segments dirty brown.

*Female*. Unknown.

#### Distribution.

N Vietnam: Vinh Phuc and C Vietnam: Nghe An.

#### Etymology.

The species is named after the country of origin: Vietnam.

##### Additional Vietnamese species

### Hartemita singaporensis

(Mao, 1945)

http://species-id.net/wiki/Hartemita_singaporensis

Figs 68–71

#### Material.

2 females (IEBR, RMNH), “Card.055”, “Card.056”, “[S Vietnam:] Dak Lak, Easo, coffee farm, MT, ? 108°37’E, Ngo Hien”; 2 males (IEBR), “Card.033”, “Card.034”, “[C Vietnam:] Thua Thien-Hue, Nam Dong, MT, 02–06.v.2005, N. Q. Truong”.

#### Notes.

All specimens from Vietnam have the ocelli small, OOL 3 times diameter of posterior ocellus (about 2.5 times in Malaysian specimens); temple narrow, transverse diameter of eye 1.1 times width of temple in lateral view; notauli narrow, smooth; scutellar sulcus with 3 cross carinae (3–5 cross carinae in Malaysian specimens); mesopleuron mainly smooth medially with sparse fine punctures; precoxal sulcus crenulate anteriorly and smooth posteriorly. New record for Vietnam.

**Figures 47–55. F8:**
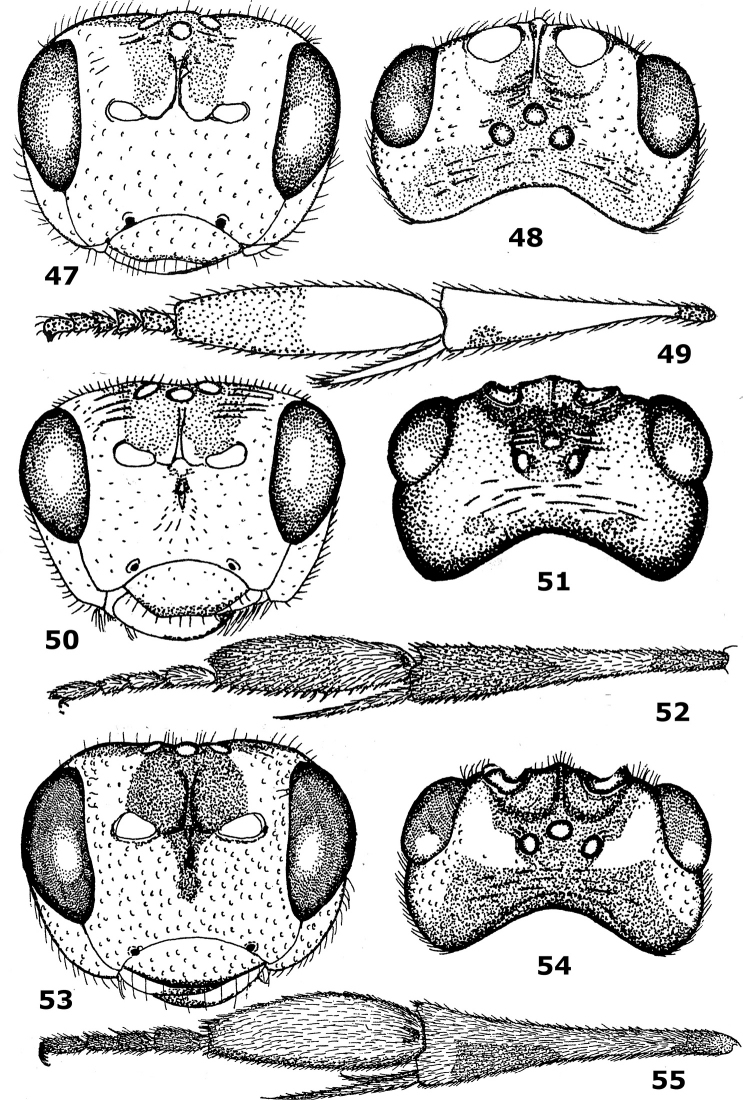
47–49 *Hartemita rudis*(Mao), female, holotype. **50–52.** *Hartemita rhadinotarsa*Dangerfield & Austin, female, paratype. **53–55.** *Hartemita basilaris*Dangerfield & Austin, female, holotype. **47, 50, 53** head frontal **48, 51, 54** head dorsal **49, 52, 55** hind tibia and tarsus lateral. After Dangerfield and Austin (1990).

**Figures 56–64. F9:**
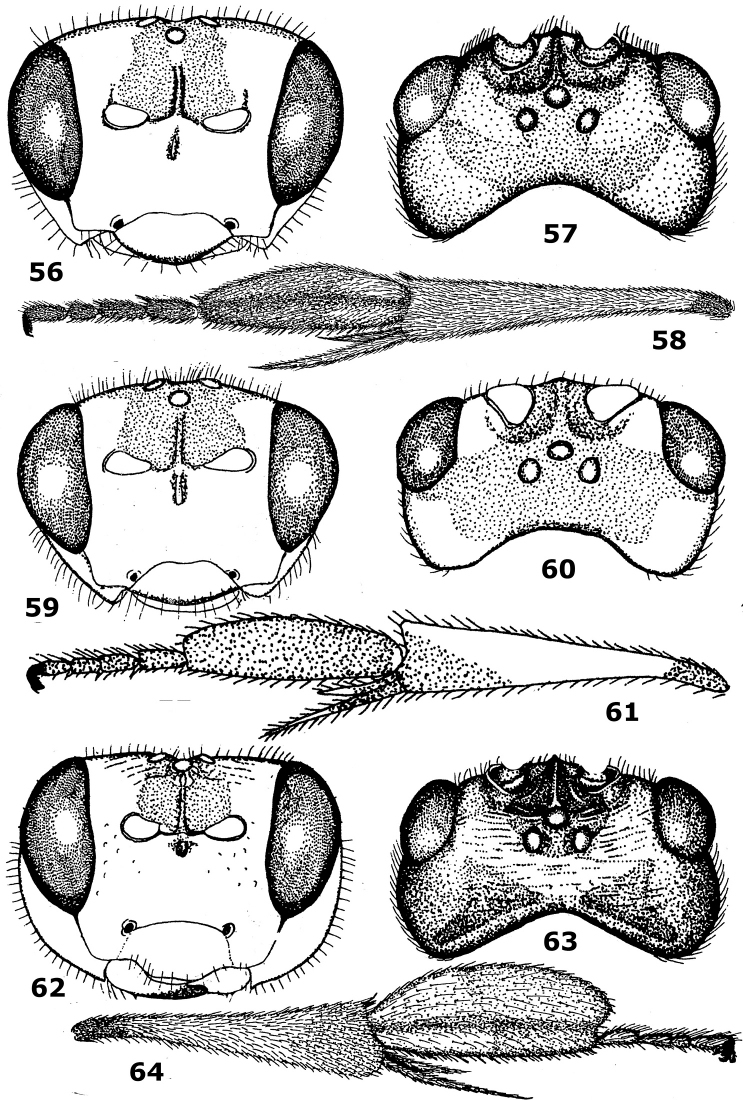
56–58. *Hartemita maculata* sp. n., female. **59–61.** *Hartemita chapini*Dangerfield & Austin, female. **62–64**. *Hartemita basilaris*Dangerfield & Austin, female, holotype. **56, 59, 62** head frontal **57, 60, 63** head dorsal **58, 61, 64** hind tibia and tarsus lateral. After Dangerfield and Austin (1990).

**Figures 65–74. F10:**
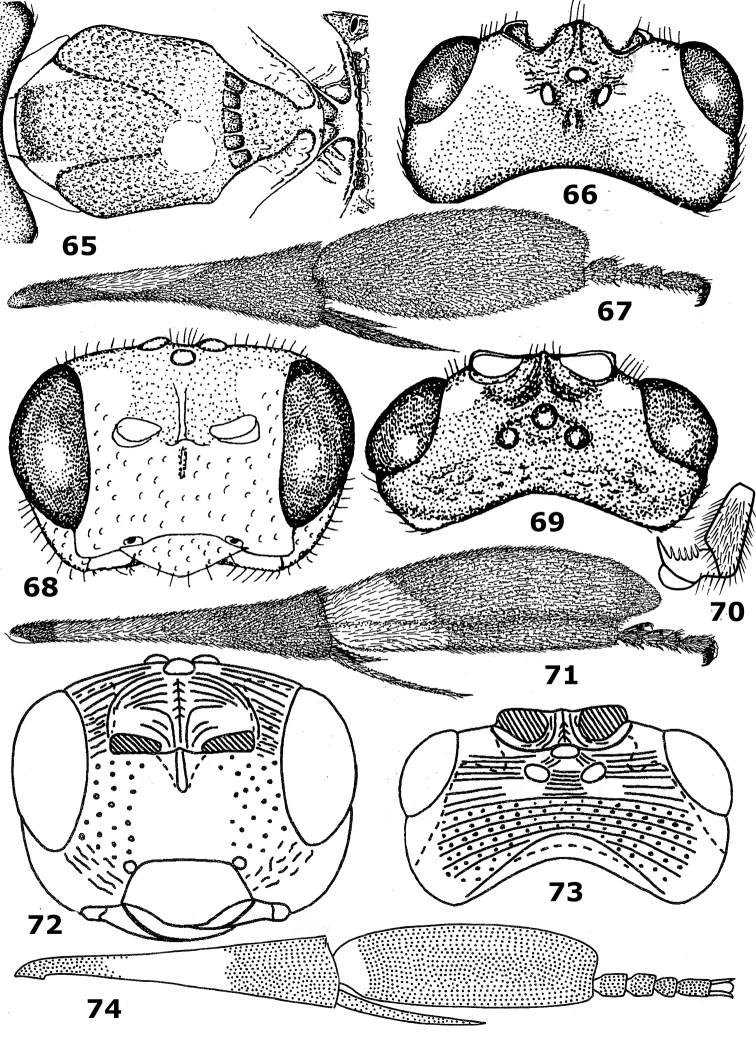
65–67. *Hartemita bruneiensis*Dangerfield & Austin, male, holotype. **68–71.** *Hartemita singaporensis* (Mao), female. **72–74.** *Hartemita spasskensis*Belokobylskij, female, holotype. **65** mesonotum and metanotum dorsal **68, 72** head frontal **66, 69, 73** head dorsal **67, 71, 74** hind tibia and tarsus lateral **70** hind tarsal claw. Figures **65–69, 71** after Dangerfield and Austin (1990) and **72–74** after Belokobylskij (2005)

**Figures 75–83. F11:**
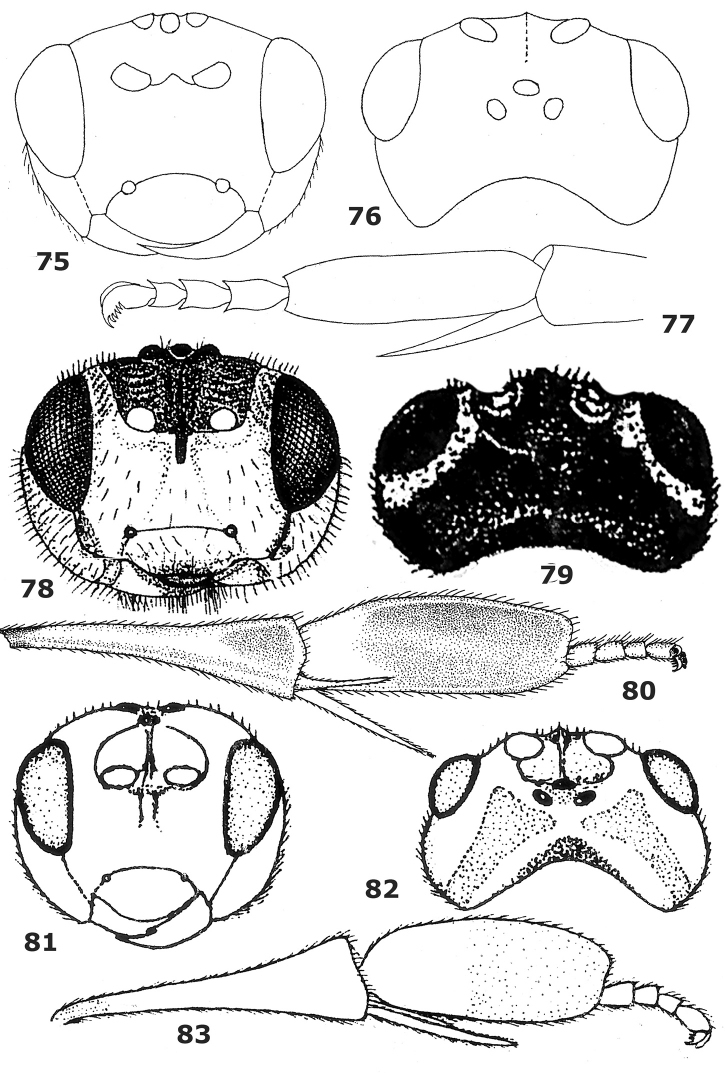
75–77. *Hartemita buteae*Ahmad & Shujauddin, male, holotype. **78–80**. *Hartemita nigrotestacea* Belokobylskij & Ku, female. **81–83**. *Hartemita flava*Chen, He & Ma. **75, 78, 83** head frontal **76, 79, 82** head dorsal **77, 80, 83** hind tibia and tarsus lateral. Figures **75–77** after Ahmad and Shujauddin (2004), **78–80** after Belokobylskij and Ku (2001) and 81–83 after Chen, He and Ma (1998)

**Figures 84–92.**
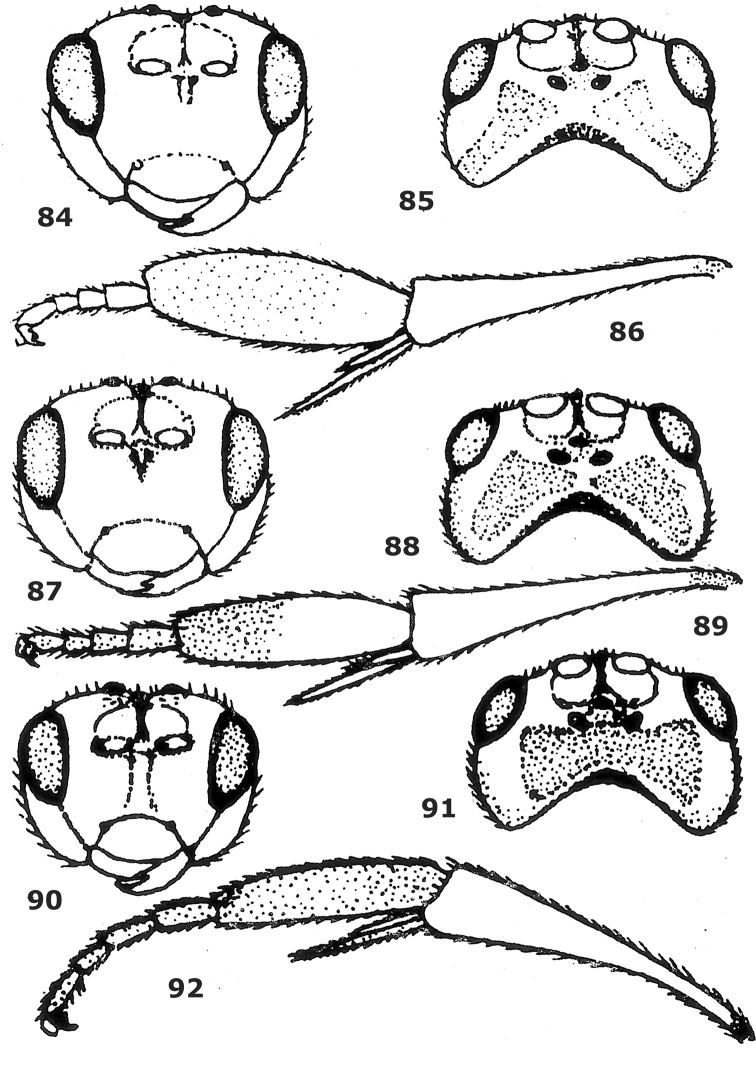
84–86. *Hartemita chinensis*Chen, He & Ma, female. **87–89**. *Hartemita excavata* Chen, He & Ma, male, holotype. **90–92**. *Hartemita punctata*Chen, He & Ma, male, holotype. **84, 87, 90** head frontal **85, 88, 91** head dorsal **86, 89, 92** hind tibia and tarsus lateral. After Chen, He and Ma (1998).

**Figures 93–96.**
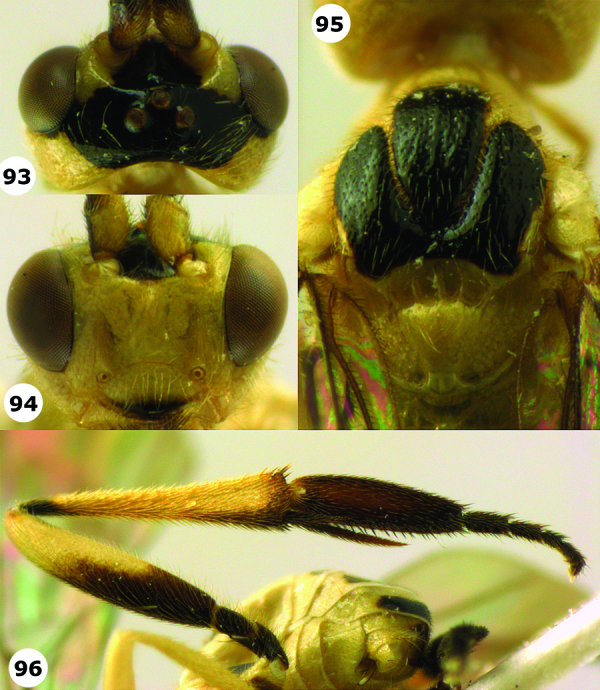
*Hartemita latipes* Cameron, male, East Malaysia (Sabah). **93** head dorsal **94** head dorsal **95** mesoscutum and scutellum dorsal **96** hind leg.

**Figures 97–100.**
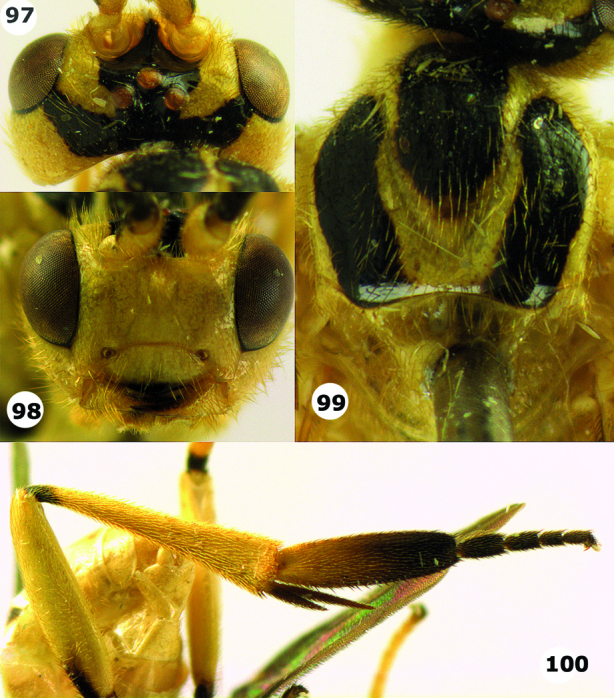
*Hartemita maculata* sp. n., male, paratype, Vietnam. **97** head dorsal **98** head dorsal **99** mesoscutum and scutellum dorsal **100** hind leg.

## Supplementary Material

XML Treatment for Hartemita

XML Treatment for Hartemita coffeana

XML Treatment for Hartemita daklaka

XML Treatment for Hartemita khuatbaolinhae

XML Treatment for Hartemita maculata

XML Treatment for Hartemita similis

XML Treatment for Hartemita vietnamica

XML Treatment for Hartemita singaporensis
